# Cyclical regression covariates remove the major confounding effect of cyclical developmental gene expression with strain-specific drug response in the malaria parasite *Plasmodium falciparum*

**DOI:** 10.1186/s12864-021-08281-y

**Published:** 2022-03-05

**Authors:** Gabriel J. Foster, Mackenzie A. C. Sievert, Katrina Button-Simons, Katelyn M. Vendrely, Jeanne Romero-Severson, Michael T. Ferdig

**Affiliations:** Eck Institute for Global Health, Department of Biological Sciences, University of Notre Dame, IN Notre Dame, USA

**Keywords:** Malaria, Transcription, *Plasmodium falciparum*, Gene expression, Confounding factors

## Abstract

**Background:**

The cyclical nature of gene expression in the intraerythrocytic development cycle (IDC) of the malaria parasite, *Plasmodium falciparum*, confounds the accurate detection of specific transcriptional differences, e.g. as provoked by the development of drug resistance. In lab-based studies, *P. falciparum* cultures are synchronized to remove this confounding factor, but the rapid detection of emerging resistance to artemisinin therapies requires rapid analysis of transcriptomes extracted directly from clinical samples. Here we propose the use of cyclical regression covariates (CRC) to eliminate the major confounding effect of developmentally driven transcriptional changes in clinical samples. We show that elimination of this confounding factor reduces both Type I and Type II errors and demonstrate the effectiveness of this approach using a published dataset of 1043 transcriptomes extracted directly from patient blood samples with different patient clearance times after treatment with artemisinin.

**Results:**

We apply this method to two publicly available datasets and demonstrate its ability to reduce the confounding of differences in transcript levels due to misaligned intraerythrocytic development time. Adjusting the clinical 1043 transcriptomes dataset with CRC results in detection of fewer functional categories than previously reported from the same data set adjusted using other methods. We also detect mostly the same functional categories, but observe fewer genes within these categories. Finally, the CRC method identifies genes in a functional category that was absent from the results when the dataset was adjusted using other methods. Analysis of differential gene expression in the clinical data samples that vary broadly for developmental stage resulted in the detection of far fewer transcripts in fewer functional categories while, at the same time, identifying genes in two functional categories not present in the unadjusted data analysis. These differences are consistent with the expectation that CRC reduces *both* false positives and false negatives with the largest effect on datasets from samples with greater variance in developmental stage.

**Conclusions:**

Cyclical regression covariates have immediate application to parasite transcriptome sequencing directly from clinical blood samples and to cost-constrained *in vitro* experiments.

**Supplementary Information:**

The online version contains supplementary material available at 10.1186/s12864-021-08281-y.

## Background

The parasite *Plasmodium falciparum* causes the most lethal form of malaria, a vector-borne disease that killed an estimated 405,000 people in 2018, 272,000 of them children under the age of five [1]. Although malaria prevention strategies and early treatment efforts have reduced the worldwide incidence of malaria by 18% since 2010, the persistence of the disease is due in part to the rapid emergence and spread of parasites resistant to antimalarial drugs, including artemisinin-based combination therapies, the last line of defense in regions where multiple drug resistance has arisen [2–4].

Most antimalarial drugs, including artemisinin-based combination therapies (ACT), target the parasite in the intraerythrocytic development cycle (IDC), when the parasite invades red blood cells [5]. However, different ACTs have reduced effectiveness at specific developmental stages during the IDC. Accurate detection of the resistant phenotype for a particular drug treatment requires accurate identification of these stages. Given accurate detection of stage within the IDC, whole transcriptome gene expression of the malaria parasite, in combination with whole genome DNA sequence, can identify pathways that result in drug resistance [6–11]. Frontline detection of newly emerging resistance to ACT therapies requires comparison of multiple single time point clinical *(ex vivo*) samples and therein lies an important problem. Unless all transcriptomes are precisely aligned at identical stages of development, changes in gene expression due to drug response are significantly confounded with gene expression due to developmental stage.

The vast majority of *P. falciparum* transcripts is expressed in a single sinusoidal pattern smoothly extending in a continuous cascade across a morphological progression through three asexual stages: ring, trophozoite, and schizont [12–15]. The period of this curve corresponds to one complete progression through the IDC and can be extended or compressed with varying amplitude corresponding to changes in maximal gene expression depending on the characteristics of individual parasite’s IDC length[16]. Importantly, the order of periodically expressed genes is broadly conserved between parasites but not entirely static [16]. This pattern is observed in other *Plasmodium* species and another apicomplexan parasite, *Toxoplasma gondii* [17]. Accurate detection of transcriptional differences between parasite strains across the IDC requires that the comparisons are made at coinciding developmental points in the IDC. For this reason, comparative analysis of gene expression in drug susceptible and drug resistant strains remains a challenge due to the potentially large confounding effects of the cyclical gene expression that drives the *P. falciparum* development through the three asexual stages of IDC [18].

While single *P. falciparum* cultures *in vitro* and infections *in vivo* in a single individual progress in a largely synchronous manner, the determinants of synchrony are not yet fully understood, influenced by both external factors and an intrinsic circadian-like regulatory mechanism [16]. For *in vitro* studies, it is possible to impose synchrony experimentally, albeit imperfectly. The point at which half of an experimentally well-synchronized culture has converted from mature schizonts to new rings, the first stage of progression through the IDC, is the 0 h post-invasion (hpi) benchmark [19–21]. Detection of this “start point” is usually accomplished visually using coarse differences in cellular morphology, e.g. between rings and schizonts. Samples are then collected at specific time increments after 0 hpi, prescribed by the particular experiment. In the case of *ex vivo* studies, the parasites taken straight from patients are not laboratory cultured and thus these samples cannot be experimentally synchronized and aligned relative to each other. Simple morphological examination of samples based on microscopy is not accurate enough to ensure that *ex vivo* transcriptomes are precisely aligned [22]. The predictive strength of the correlation of gene expression values to a measure of drug resistance for each *ex vivo* sample (e.g., patient clearance half-life) depends heavily on minimizing the confounding effect using an alignment method that can account for the cyclical pattern of stage progression.

The emergence of resistance to artemisinin therapies, the last line of defense in geographic regions where multiple drug resistance has arisen, occurred during the time when rapid whole transcriptome sequencing of parasites in *ex vivo* samples became feasible [23, 24]. This spurred an intensive search for the genetic mechanisms of artemisinin resistance through comparison of parasites transcriptomes in *ex vivo* blood samples taken from patients for whom drug clearance half-lives, the standard clinical measure of artemisinin resistance, were also available. While *P. falciparum* exhibits synchronous behavior within a single person, blood samples taken from different people will necessarily be at some degree of different IDC developmental stages. In a study of 1043 *ex vivo* whole transcriptome samples designed to detect the mechanisms for artemisinin resistance, Mok et al. relied on k-means clustering as a transcriptional staging method, identifying three subgroups of samples based on their developmental profiles [24]. Comparison of the three clusters of whole transcriptomes against the multi-time point transcriptome data of parasite strain Dd2 yielded transcriptional stage estimates of 8-10 hpi for Group A and a much broader transcriptional stage distribution (10-20 hpi) for group B [24, 25]. The authors concluded that the three subgroups (A, B and C) mapped onto the three stages in IDC progression, with the best fit for Group A and the worst for the late stage group C, which was not extensively analyzed.

IDC progression differences within these clusters could still lead to misattribution of stage-based transcriptional differences to the variable under study (e.g., drug susceptibility). Another study applied a novel correction method to the 2015 Mok et al. data by removing the effect of a linear and polynomial covariate of IDC time point before differential expression analysis [26]. Because segments of the broadly sinusoidal progression curve can be approximated by linear and polynomial functions, that method improves accuracy in sample sets with small variance in IDC progression. However, as the method does not specifically incorporate the cyclic nature of expression, it is prone to potential overfitting and is not useful for samples whose developmental stage distribution spans reinvasion of new uninfected RBCs as the developmental cycle continues.

Cyclical regression covariates have been applied to a range of biological data with cyclical covariance, including diarrheal severity, transmission of malaria, and seasonal changes in human gene expression [27–32]. Here we examine the use of cyclical regression covariates to disentangle expression differences due to developmental stage progression from differences in response to clinical artemisinin treatment in *P. falciparum* parasites. We demonstrate that small differences in developmental stage can generate both false positive and false negative associations between transcript levels and the clinical phenotype for artemisinin resistance and show how these errors can be significantly reduced by using CRC to align developmental progression in two different strains. We then apply this method as a linear model correction with cyclical regression covariates to the Mok et al. set of *ex vivo P. falciparum* transcriptional samples previously assessed by transcriptional staging against Dd2 and found fewer differences in the number of transcripts identified as differentially expressed in clinically artemisinin resistant parasites[24]. The genes we identified as differentially expressed were largely a subset of the genes identified in the Mok et al. 2015 study. However, we also detected differentially expressed genes in a functional category not detected in the Mok et al. 2015 study. Our results, taken in combination with our models and simple demonstrations with real data, indicate that CRC successfully reduces the number of false positives and false negatives that result from misalignment of developmental stages.

## Results

### IDC misalignment generates type I and type II errors

**Fig. 1:**
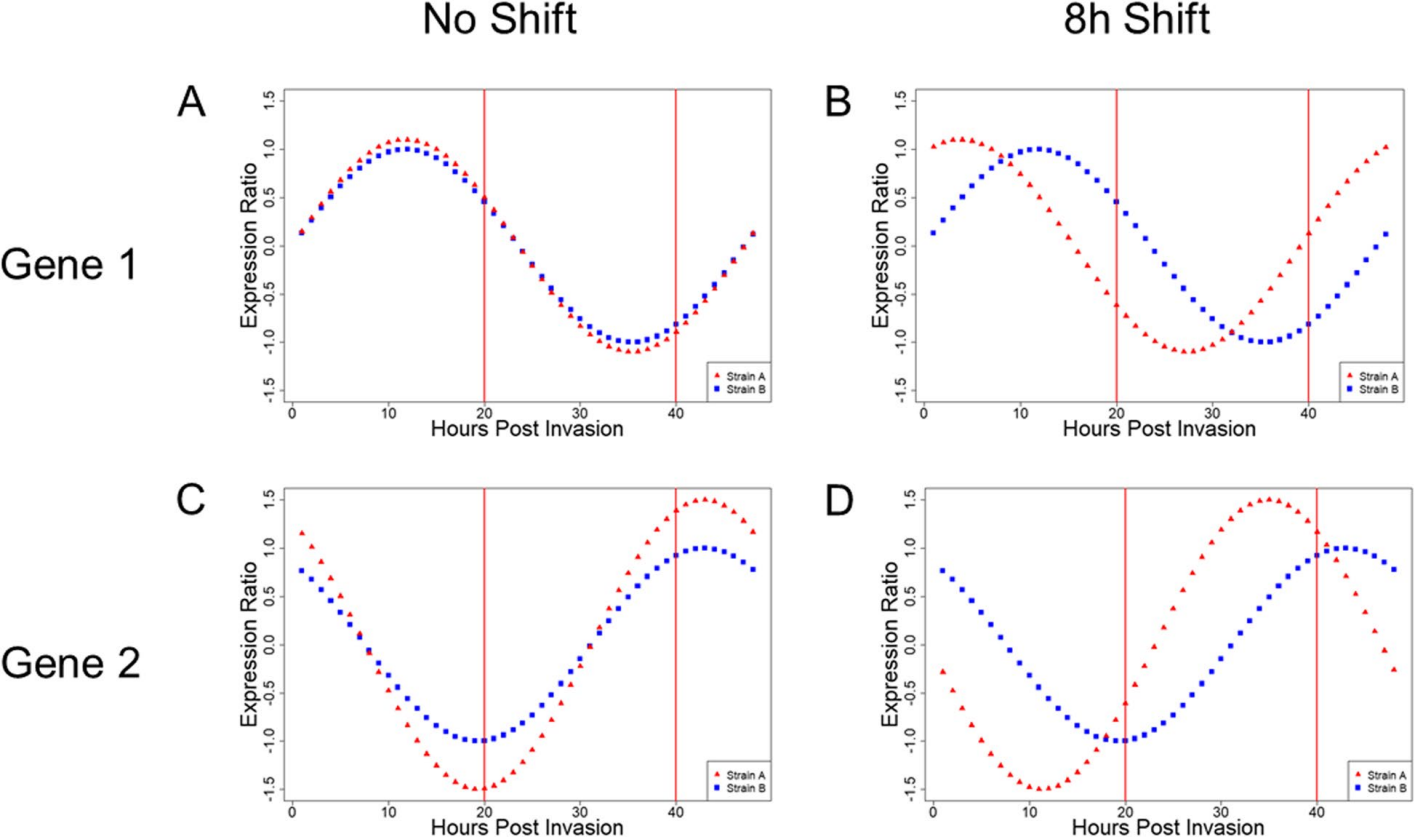
Errors caused by comparison of improperly aligned cyclical transcription data. **A** and **B** show two model features with identical expression patterns; **C** and **D** represent two model features with real differences in gene expression. **A**: When 0 hpi is correctly aligned for both, single observations (red bars) correctly detect no difference. **B**: When 0 hpi is improperly aligned, single observations (red bars) detect a difference between strains where none exists. **C**: Proper alignment of 0 hpi allows single observations (red bars) to detect the true difference. **D**: Improper alignment of 0 hpi obscures the true difference in single observations.

Our simple model has two different *P. falciparum* strains with identical gene expression profiles. Comparison of a transcriptional sample at the same point in developmental progression reveals no differential expression (Fig. 1 A). However, when the two strains are sampled at different points in their respective cyclical transcriptional progression and compared without adjustment, a significant gene expression difference is falsely detected, a Type I error (Fig. 1B). Two strains with a true gene expression difference sampled at the same point in IDC progression will show a significant difference that actually exists (Fig. 1 C). Sampling these same two strains at different time points in their respective progressions can generate the appearance of a difference where none exists (a Type I error) while failing to detect a true difference, a Type II error (Fig. 1D).

**Fig. 2:**
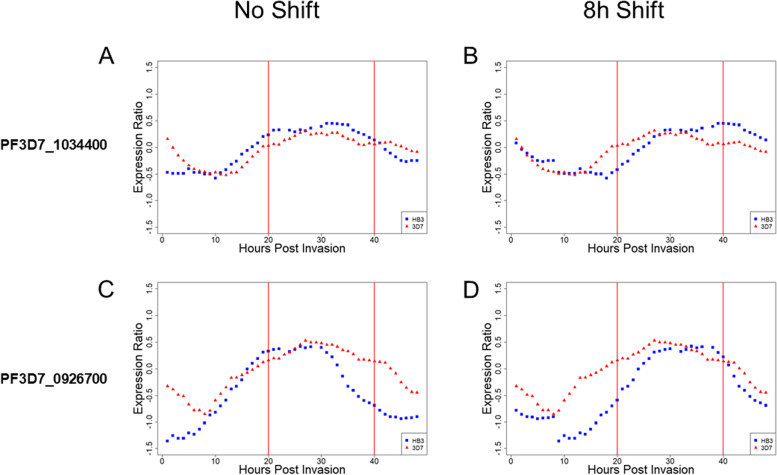
Comparison of *P. falciparum *transcriptional profiles of two features in strains HB3 and 3D7 data.** A**: Correct assignment of 0 hpi shows little difference in the expression of PF3D7_1034400 between HB3 and 3D7. **B**: An 8 h shift in HB3 IDC progression timing produces differential expression at 20 h and 40 h due to the progression shift. **C**: In PF3D7_0926700, a genuine expression difference exists between HB3 and 3D7 at 40 hpi. **D**: An 8 h shift in HB3 expression obscures the differential expression at 40 h, and generates a difference at 20 h; the misalignment creates both Type I and Type II errors. Figures **A**, **B**, **C**, and **D** are derived from transcriptional time course data from HB3 and 3D7 [15, 33].

Examination of the transcriptional profiles of two features (PF3D7_1034400 and PF3D7_0926700) in the *P. falciparum* strains HB3 and 3D7 illustrates the effect of misalignment in real data [15, 33]. The sequence PF3D7_1034400 displays a characteristic wave pattern of expression across the entire IDC that is similar for both strains when transcriptional profiles correctly begin at 0 hpi (Fig. 2 A). A computational shift of the sequence from HB3 eight hours forward results in the appearance of different expression between the two sequences across multiple time points, false positives driven solely by misalignment (Fig. 2B). In the alternative case, sequence PF3D7_0926700 shows clear transcriptional differences at 40 hpi between HB3 and 3D7 when t = 0 hpi is correctly determined for both parasite strains (Fig. 2 C). Computationally shifting HB3 eight hours forward renders this transcriptional difference at 40 hpi undetectable (a Type I error) and generates a false difference at 20 hpi (a Type II error) (Fig. 2D).

### Performance of cyclical regression covariates when IDC progression is the only variable

We tested the effect of cyclical regression covariates on a published *P. falciparum* dataset containing transcriptional profiles of parasite strain PB58 collected at three time points (6 hpi, 26 hpi, and 38 hpi) with three biological replicates for each time point [34]. Because all of the samples were taken from the same parasite and the data were replicated, any differential expression detected must solely be a result of different IDC progression times. Pairwise comparison of different time points (e.g., 6 hpi with 26 hpi) reveals the extent of possible false positive associations when the confounding effect of IDC progression is not removed (Table 1. The large number of genes considered significantly expressed entirely as a result of progression differences demonstrates that any stage-blind analysis of transcription risks large confounding effects due to distinct stage progressions.

**Table 1:** Removing the effect of stage removes Type I errors. Three replications of whole transcriptome samples from PB58 at 3 time points; 6 hpi, 26 hpi, and 38 hpi. A: *In silico* misalignment of the time points reveals the extent of expression differences that appear due to this misalignment (p < 0.01, FDR < 0.05). B: Application of cyclical regression covariates to correctly align the replications results in no differences in gene expression, the expected result of replication for a single strain in the haploid IDC stage

		**Original samples**		
		6 hpi	26 hpi	38 hpi
***In silico*** **mis-aligned samples**	6 hpi	-	2989	294
	26 hpi	2989	-	457
	38 hpi	294	457	-
**CRC aligned samples**	6 hpi	-	0	0
	26 hpi	0	-	0
	38 hpi	0	0	-

We next generated a differential expression analysis using a linear model including the covariates (Methods). Briefly, if we consider IDC progression as a circle, we can calculate any sample’s location on that circle using its IDC time period and include those coordinates as covariates in a linear model. After accounting for stage progression using cyclical regression covariates, our analysis finds no differentially expressed genes. (Table 1) The method eliminated the confounding that caused the false positives in this dataset.

### Application of cyclical regression covariates to a set of *ex vivo* P. falciparum transcript samples

**Fig. 3:**
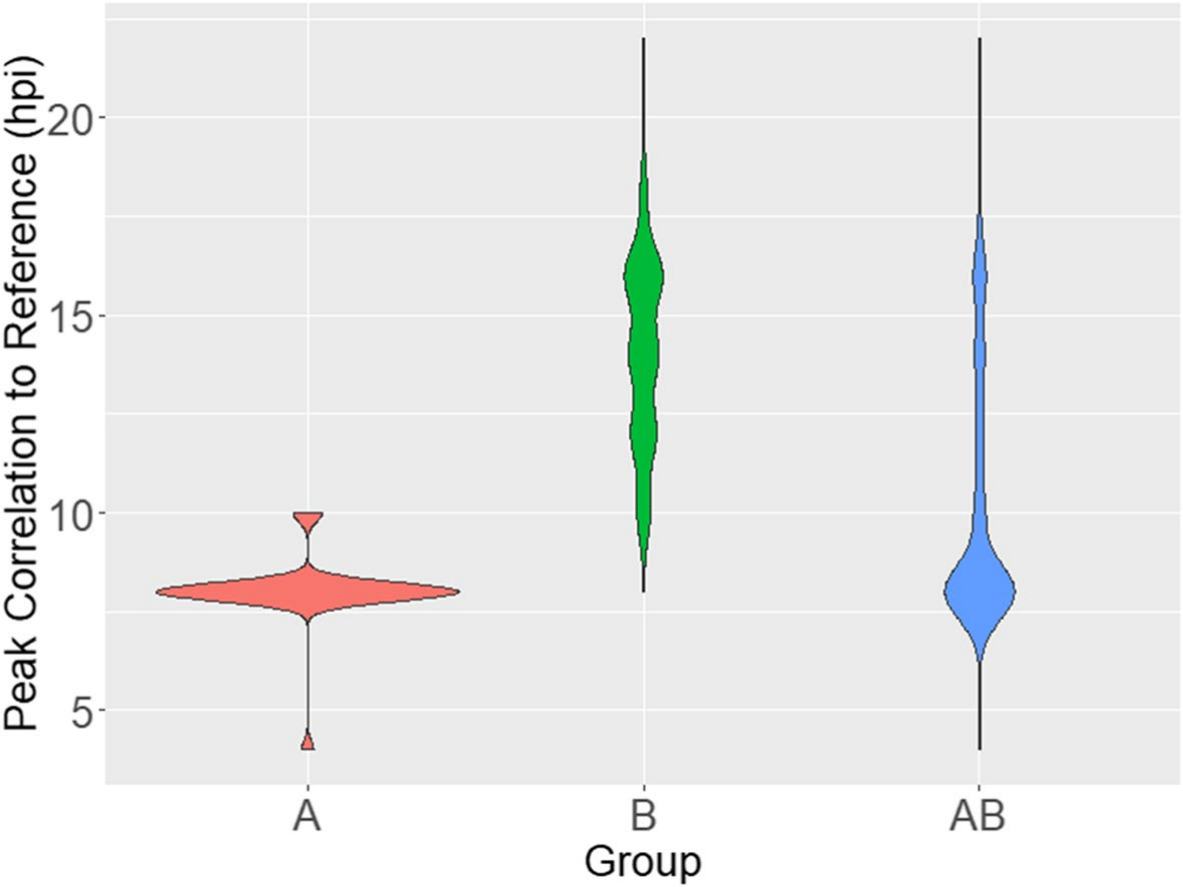
Stage Distribution of Mok et al. 2015 data. Distribution of the maximum correlation of each sample to each time point in the Dd2 reference time course in hours post invasion (hpi) [25]. The distribution in Group A is relatively narrow, indicating good alignment by developmental stage; the distribution in Group B indicates poor alignment. Group A+B is the A and B samples combined, then aligned. The A+B analysis was done to test the ability of CRC to detected non-developmental stage related expression differences in poorly staged samples.

Comparison of transcriptional profiles of *ex vivo* samples could reveal the genetic basis for known drug resistance and reveal new resistance mechanisms; however, the transcriptomes of parasites from these clinical samples will necessarily represent a range of different stages of progression through the IDC. A confounding factor, in this case progression through the IDC, is a variable that influences the values of another variable, in this case response to artemisinin. If the approach used to identify Group A has resulted in a relatively accurate alignment of developmental stage across samples (Fig. 3), we expect that when we use the inferred IDC progression time calculated by the authors, our correction method will result in the nearly the same set of differentially expressed genes. Because Group B samples represent a broader stage distribution (10-20 hpi) (Fig. 3), we expect that the gene expression analysis of uncorrected versus corrected data will result in much less overlap.

**Fig. 4:**
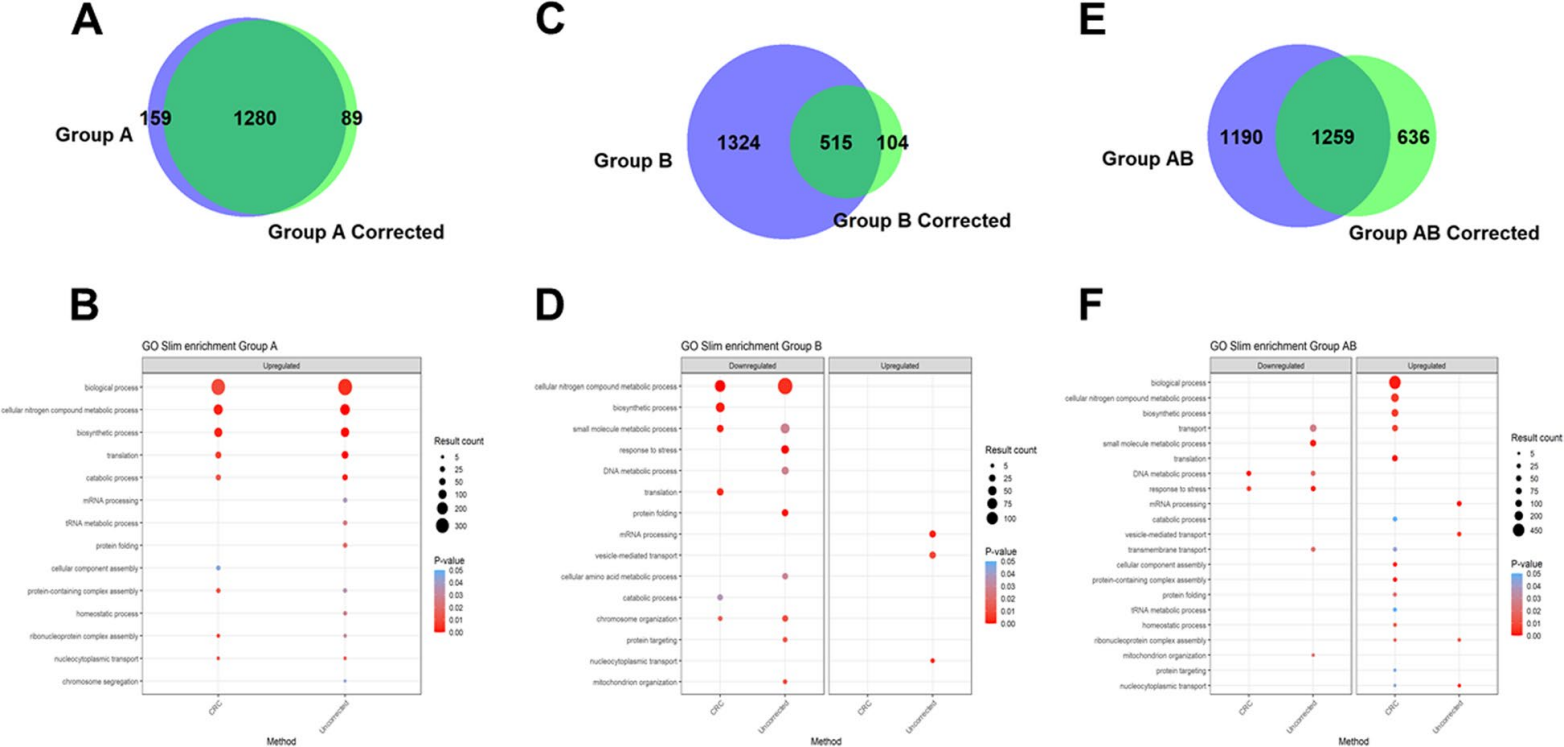
Overlap of genes considered significant before and after correction with GO Slim results. Comparison of the genes considered significantly correlated with patient clearance half-life in Groups A (**A**), B (**B**) and A+B (**C**) before (green) and after (purple) the application of cyclical regression covariate correction. **D**, **E**, and **F** show the comparison of up and downregulated GO Slim categories before and after the application of cyclical regression covariate correction for each group A, B, and A+B, respectively. Size of the point corresponds to the number of genes detected in the particular category and color corresponds to P-value.

The Venn diagram for the genes identified as differentially expressed in Group A shows an 83.8% overlap (Fig. 4 A). No genes are identified as downregulated in Group A in either case (Additional Files 3 and 4). Examination of the differentially expressed upregulated genes by Gene Ontology (GO) Slim subsets reveals that the uncorrected data contain five GO Slim subsets (a total of 69 genes) not detected in the corrected Group A, while one GO Slim subset ‘Cellular component assembly’ containing 17 genes, is absent from the uncorrected analysis (Fig. 4, Additional File 3). An investigation of these 17 genes reveals the inclusion of two additional proteasome subunits PF3D7_0723600 (proteasome assembly chaperone 4, putative) and PF3D7_1130400 (26 S protease regulatory subunit 6 A, putative). Protein homeostasis and the proteasome have been implicated in resistance as a key target for artemisinin mediated killing [35, 36] and drug response [37]. Proteasome inhibitors are highly synergistic with artemisinin and reverses artemisinin resistance making the proteasome an important target for combination therapies and drug development [38–40]. The addition of these 17 genes agrees with and further bolsters the original findings presented in Mok et al. and adds new features that merit additional investigation.

Comparison of the results of this same analysis conducted on Group B genes reveals a much larger difference between the uncorrected and corrected analyses, with only a 26.5% overlap (Fig. 4 B). The uncorrected Group B analysis identifies upregulated genes in three GO Slim subsets totaling 59 genes, while no upregulated genes are detected in CRC corrected Group B. The uncorrected Group B analysis identifies 395 downregulated genes across nine GO Slim subsets, while the corrected Group analysis identifies many fewer downregulated genes (219) across six GO Slim subsets, three of which are shared with the uncorrected analysis (Fig. 4 E, Additional File 3). This relative degree of overlap (or lack of it) in Groups A and B is expected, assuming that better alignment of stage across samples will reduce the number of false positives as well as identify what were false negatives in the uncorrected analysis. Using our CRC adjustment, we have generated data upon which to base new, more precise, testable hypotheses of a gene list less likely to include both false positives and false negatives.

The results of our functional analysis of Group B genes highlight another potential value of the CRC method. Mok et al. did not include an investigation of Group B in their analysis and conclusions, presumably because of their estimated broad distribution of developmental stages ranging from 10 to 20 hpi and their expectation that later stages do not differ in artemisinin response between resistant and sensitive parasites. However, a slowing of the transcriptional program during this later ring stage of the IDC has been associated artemisinin resistant parasites [23]. Our GO enrichment analysis of the genes in Group B found GO Slim categories of ‘Translation’, ‘Chromosome organization’, and some metabolic processes, suggesting the gene expression for these late ring to early trophozoite samples have relevant information about artemisinin resistance and related phenotypes.

We further challenged our method by combining the Mok et al. *ex vivo* data, creating Group A+B, to simulate the scenario wherein samples are broadly poorly staged (i.e., as would occur when there is not an option to focus on a precisely staged set) (Fig. 3, Additional File 3). The combined analysis found an overlap of 40.8% between corrected and uncorrected Group A+B (Fig. 4C). The corrected Group A+B analysis identified 1051 genes upregulated in 15 GO Slim subsets, many more than the 695 upregulated genes when Group A was analyzed alone. Importantly, following CRC correction of Group A+B, 15 upregulated GO Slim categories were identified, including all 9 from the CRC-corrected Group A GO Slims, with the categories occurring in the same ranked order of significance (with no correction applied, we find 2 of 9 GO Slims) (Fig. 4 F, Additional File 3). The A+B analysis detected 72 downregulated genes in two GO Slim subsets (‘DNA metabolic process’ and ‘Response to stress’) that were not detected in the CRC-corrected analysis of Group B alone. Notably, these two GO Slim subsets were detected as downregulated in Group A+B without CRC applied. While a downregulation of ‘DNA metabolic processes’ was reported as enriched in the original Mok et al. study supporting our observation, we also find the inclusion of downregulated ‘Response to stress’ in our analysis (a function that was removed from Group B after CRC correction). The re-inclusion of ‘Response to stress’ diverges from the corrected analysis of Group B and other reports that artemisinin causes oxidative stress through damage of proteins. We therefore conservatively interpret this observation as a false positive; CRC correction may not overcome an excess of poorly aligned developmentally staged samples, as simulated by our Group A+B analysis. Application of CRC correction should be used in concert with rigorous experimental methods to control for developmental stage composition among the samples when possible.

## Discussion

Precise alignment of *P. falciparum* developmental stages improves the accuracy of detection of transcriptional changes in the parasite due to specific perturbations. The challenge of accurately aligning stage can be partly assuaged when working in cultured (*in vitro*) parasites using synchronization methods and careful monitoring of stage composition, however these steps are time and labor consuming and some stage variation will remain. For studies using clinically derived (*ex vivo)* blood samples it is not possible to experimentally control for the stage composition. Studies that aspire to efficiently identify emerging drug resistance mechanisms using transcript readouts will benefit from a method that can specifically correct for diversity in developmental stages among clinical samples. A specific (known) confounding factor cannot be removed post hoc by simply changing the False Discovery Rate to zero or lowering the value used to decide the cutoff for significance. Such a strategy is based on the fundamental assumption that the investigator has already accounted for serious confounding in the experimental design or has validated a method of correction prior to analysis. Here we demonstrated how misalignment of IDC between samples generates both Type I and Type II errors. We further demonstrated the strong impact that even a small degree of misalignment has on the detection of differential gene expression associated with drug resistance.

We expected to see an impact of CRC on our reanalysis of the Mok et al. 2015 data because of the ideal opportunity it provided to directly assess our method on samples where stage could not be experimentally controlled. While we detected small differences even in the comparatively well-staged Group A, we observed much larger differences between CRC-corrected and CRC-uncorrected in Group B. Importantly, the Gene Ontology enrichments of the differentially expressed genes we detected in Group B contain many terms associated with cellular metabolism, a finding consistent with the working theory that Kelch13 propeller mutations influence rates of endocytosis [41]. The lack of free heme produced by a reduction in hemoglobin uptake and digestion would, in theory, limits the activation of artemisinin. However, reducing parasite metabolism may produce broader effects on parasite biology in the form of a slower cell cycle progression from late rings to trophozoites. Our observation of downregulated GO Slims, ‘Translation’ and ‘Chromosome organization’, are consistent with the parasite slowing its development through transcriptional mechanisms. With the use of CRC, we find that analysis of group B provides offers additional interpretation than was presented in the original study.

Most transcribed genes are strongly influenced by developmental stage. Their expression across the cycle takes the form of a cascade that cannot be accurately parsed using visual assignment of morphological stages [15, 16, 33]. A recent study of the transcriptional cascades of parasites with varying cycle lengths demonstrated a broad consistency in the order of cyclically expressed genes through the cascade, and this was not altered by overall cell cycle duration or the proportional length of specific IDC stages determined morphologically [16]. That study hypothesized an intrinsic oscillator mechanism that controls the transcriptional network that dictates the cascade and progression through the IDC [16]. The broad consistency and cyclical nature of the cascade is why the simple and intuitive CRC method can effectively correct for transcriptional differences due entirely to stage. We anticipate the CRC method can be usefully extended to *in vitro* time series data, investigations of mechanisms controlling the cascade, and the regulation of transcription as pertains to artemisinin resistance and the connection of cell metabolism to transcriptional signatures of stage progression at high resolution. With the exception of single cell RNAseq studies, any statement about a *P. falciparum* sample’s location in the IDC is, in reality, an estimate of the midpoint of the IDC progression of all parasites in an *ex vivo* sample. The general assumption is that *in vivo*, within a single human host, parasites are highly synchronized. This assumption rests on years of clinical data that show that the recurrent fever occurs when millions of parasites simultaneously burst out of circulating red cells. Novel resistance phenotypes may involve a degree of asynchrony with the result that some parasites escape drug treatment targeted to the early ring stage. Reports of patients with two parasite broods shifted by 24 h, instead of one brood synchronized to a 48 h cycle indicate that the parasite is capable of altering the period of the cycle [42]. Different parasite strains infecting the same individual may not be synchronous with each other, resulting in a complex pattern of overlapping developmental cycles that our simple method does not address [43]. Predictive modeling based on RT-qPCR of only three genes verified as markers of developmental stage, combined with patient symptoms, can be used to identify parasite age in single clone infections [44]. Our method along with other improved methods for direct detection of parasite stage will enhance the ability of the malaria community to extract meaningful data from clinical samples in all stages in the IDC, especially in geographic regions where single clone infections are typical. This is the case for the Mekong delta and surrounding regions, where parasites resistant to artemisinin and ACT therapies have originated and continue to spread. Reports continue to illustrate that artemisinin resistance mechanisms are neither simple nor fully elucidated putting ACT therapies at risk [41]. The straightforward CRC method, by reducing the false positives generated by misaligned developmental stage progression, is a step toward a more precise understanding of the mechanisms of resistance artemisinin and other antimalarial drugs.

## Conclusions

Here we have demonstrated the use of cyclical regression covariates to reduce the major confounding factor in studies focused on the detection of expression difference between strains *of P. falciparum* by leveraging the unusual periodicity of transcription due to developmental progression through the IDC. Cyclical regression covariates have immediate application to studies where *in vitro* synchronization of all samples to the same developmental time point is not feasible, primarily parasite transcriptome sequencing direct from clinical blood samples, a widely used approach to frontline detection of emerging drug resistance.

## Methods

### Demonstration of the effects of progression misalignment

In order to demonstrate the effect of shifts in IDC timing on gene expression, two model data sets were used. The model datasets (Genes A and B) are sinusoidal curves with a period of 48 h. To demonstrate the effect of shifts in IDC timing in real *P. falciparum* time course data, we obtained multiple time point transcription data from PlasmoDB.org for two strains, 3D7 and HB3. [15, 33] The genes used as examples (PF3D7_1034400 and PF3D7_0926700) were chosen based on their representation of cyclic expression and their similarity (PF3D7_1034400) or difference (PF3D7_0926700) between strains. Unadjusted data was graphed as-is; adjusted data was plotted against the actual sampling time plus 8 h, with any time point past 48 h being plotted as the time point minus 48 in order to account for the 48-hour IDC.

### Determination of IDC Timing Using Transcriptional Profiles

For determination of the actual point in IDC progression for a given sample, Pearson Correlation Coefficients (PCC) between each sample’s whole transcriptome profile and the profile of each time point in the 3D7 reference IDC time course (hourly time points in the *in vitro* 3D7 IDC) were determined [23, 33]. The hours post-infection time point (hpi) corresponding to the maximum PCC was considered the sample’s IDC timing, and was used in subsequent steps. This was performed using the function *stagingByTranscription()* in the R package **PFExpTools**.

### Determination of Cyclical Regression Covariates

With samples taken at different points in progression displaying differential gene expression based solely on progression, it is necessary to identify and remove any cyclic effect in a sample set. With a linear effect, we could use a single covariate related to the confounding linear effect to correct; as our confounding effect is cyclical, we require two covariates. We calculated paired cyclical regression covariates for each transcriptional sample to correct for progression’s effect on gene expression. The progression time calculated previously (T) was used in the following formula:

$$cos(2\pi T/48),sin(2\pi T/48)$$

The covariates were generated using the function *CRCgeneration()* in the R package **PFExpTools**.

### Initial Application of Cyclical Regression Covariates

For testing the initial proof of concept, we looked for a dataset containing multiple replicated time points from a single parasite strain; this allowed us to associate any observed differences between time points as progression driven. The gene expression data used to demonstrate the efficacy of the cyclical regression covariate method were obtained from the Gene Expression Omnibus; the data for PB58 are listed under Accession Number GSE119514. We used *lm()* in R; for the uncorrected analysis our model was Timepoint ~ Gene Expression for each gene, and for the corrected analysis the model was Timepoint ~ Gene Expression + xcovariate + ycovariate. Covariates were generated as described previously. Genes were considered differentially expressed if the p-value of fit was < 0.01 and FDR < 0.05.

### Application of Cyclical Regression Covariates on a Large *ex vivo* Dataset

We used the Mok et al. 2015 *ex vivo* dataset, 1043 clinical samples taken at various points in IDC progression, to examine the effect of cyclical regression covariates on the results of the gene expression analysis. We retrieved the normalized uncurated Mok data from the Gene Expression Omnibus, Accession Number GSE59097, and updated the curation to the current transcriptome assembly. We aligned the sequence for each probe with Version 46 of the *P. falciparum* transcriptome. Probes that had an alignment with a bitscore > 130 and no secondary alignment scored over 60 were named to the gene they aligned with [45]. In the case that multiple probes aligned to a single gene, the signals were averaged. Genes with data present in greater than 80% of samples were used in the analysis. This curation was performed using the fullCurate() function in the R package PFExpTools (Additional File 1, Additional File 2).

Gene expression analysis for two groups identified by Mok et al. 2015 (Group A+B) was performed as follows. The relationship of the expression of each gene with patient clearance half-life was evaluated using a simple linear regression model in R. For each gene in the corrected dataset, the simple linear model included the paired cyclical regression coefficients. Each gene in the uncorrected dataset was evaluated using a simple linear regression model without the paired cyclical regression coefficients. For the uncorrected analysis our model was Phenotype ~ Gene Expression for each gene, and for the corrected analysis the model was Phenotype ~ Gene Expression + xcovariate + ycovariate. The cutoff value of p < 0.05 and FDR < 0.25 were the criteria for a decision of differential gene expression for each gene. The covariates were generated using the function *CRCgeneration()* in the R package **PFExpTools**; the IDC progression value provided in the associated metadata was used. We compared the gene expression analysis uncorrected and then corrected using CRC for groups A and B separately.Data analysis and figure generation were performed in R Version 3.3.1. Venn diagrams were created using the R package **VennDiagram** [46].

### GO and GO Slim annotation

We used the GO and GO Slim applications implemented in PlasmoDB beta release 50 (https://plasmodb.org/plasmo/app/). GO Slim provides a less detailed annotation by grouping features into broader categories of biological functions. This is useful when the investigators wish to focus on a given broad biological function or, as is the case with this investigation, to determine if a broad category of biological functions was missed if the CRC correction is not used. Additional files contain the Go Slim annotations (Additional File 3) and the more detailed GO annotations (Additional File 4), both with and without the CRC correction.

### Availability of data and materials

The HB3 and 3D7 time course data is available for download at PlasmoDB.org. The PB58 data is available at the Gene Expression Omnibus under Accession Number GSE119514. The Mok et al. dataset is available at the Gene Expression Omnibus under Accession Number GSE59097. The updated curation of the Mok et al., 2015 dataset is provided as Additional File 1, with patient sample labels in the columns and feature names in the rows. A figure demonstrating a quality control comparison of our curation of the Mok et al. dataset and their reported analysis is provided as Additional File 2. The R package **PFExpTools** is freely available for download and use at https://github.com/foster-gabe/PFExpTools, including all documentation and source code under the GPL-3 license. This manuscript was prepared using Version 1.0 of **PFExpTools**.

## Supplementary Information

**Additional file 1.** The result of our re-curation of the Mok et al. data originally sourced at the Gene Expression Omnibus at Accession Number GSE59097. The dataset was curated as described in Methods using the **PFExpTools** R package. This file is a matrix of expression values, with the features in rows and samples in columns in a comma delimited file format.

**Additional file 2.** Provides quality control comparisons between our curation and analysis of the Mok et al. data and the originally published results.

**Additional file 3.** Contains the Gene Ontology Go Slim category results for all the comparisons.

**Additional file 4.** Contains the complete Geno Ontology category results for all the comparisons.

## Data Availability

The HB3 and 3D7 time course data is available for download at PlasmoDB.org. The PB58 data is available at the Gene Expression Omnibus under Accession Number GSE119514. The Mok et al. dataset is available at the Gene Expression Omnibus under Accession Number GSE59097. The updated curation of the Mok et al. dataset is provided as Additional File (1) A figure demonstrating a quality control comparison of our curation of the Mok et al. dataset and their analysis is provided as Additional File (2) The R package **PFExpTools** is freely available for download and use at https://github.com/foster-gabe/PFExpTools, including all documentation and source code under the GPL-3 license. This manuscript was prepared using Version 1.0.0 of **PFExpTools**, available under DOI 10.5281/zenodo.3731280.
