# Supplementary material for: Cyclical regression covariates remove the major confounding effect of cyclical developmental gene expression with strain-specific drug response in the malaria parasite Plasmodium falciparum
Source: BMC Genomics. 2022 Mar 5;23:180. doi: 10.1186/s12864-021-08281-y (PMC8897900; doi:10.1186/s12864-021-08281-y)
Supplement: Supplementary file 2 — Additional file 2. Provides quality control comparisons between our curation and analysis of the Mok et al. data and the originally published results. [file 12864_2021_8281_MOESM2_ESM.pdf]

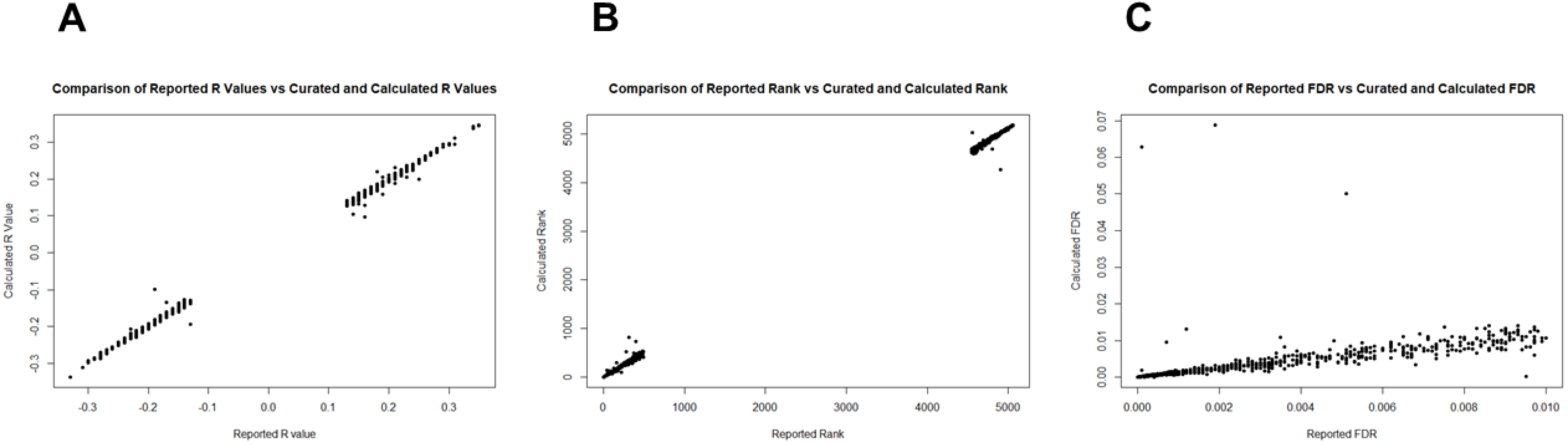

Supplemental Figure 1: Quality Control Testing of the PFExpTools Curation Functionality. The original Mok et al. study provided a list of genes considered significantly correlated with patient clearance half-life and associated statistics; we replicated the analysis on our curation of the probe-level data provided at the Gene Expression Omnibus. With very few exceptions, our R values (1A), correlation ranks (1B), and reported FDR values (1C) match the published results.
